# Spatial Plans as a Critical Intervention in Improving Population Health. A Discourse Arising from a Health Census Review of the State of Current Local Planning Policy in England

**DOI:** 10.1177/08854122241229565

**Published:** 2024-02-04

**Authors:** Michael Chang, Neil Carhart, Rosalie Callway

**Affiliations:** 1 Faculty of Engineering, University of Bristol, Bristol, UK; 2 Town and Country Planning Association, London, UK; 3 Bristol Medical School, University of Bristol, Bristol, UK

**Keywords:** spatial planning, local plans, public health, census, healthy planning

## Abstract

Planning decisions are dependent on the strength of regulatory instruments. The local plan is a public policy document informing decisions on land-use developments that can have impact on health of future populations. The article reports on a census review of local plans (n = 346) in England. Using content analysis, we found limited resilience with only 126 (36.4%) have links to local health needs, 101 (29.2%) have links to local health strategies, and 129 (37.5%) have a health policy. It is a novel approach to identify the current state of local policies as the starting point for projecting future health outcomes.

## Introduction

The planning system influences the future strategic direction, coordination and delivery of urban development activities in towns and cities. Within this system of local spatial planning in England, the local plan is created then implemented to enable positive land use decisions to be made in alignment with social, economic and environmental values of national and local authorities (Department of Health 2015). So the local plan, in effect, helps creates the conditions for deriving the benefits that decisions can provide to future populations, including health and wellbeing outcomes through the planning system. This article makes a contribution to the theme of ‘Alternative Planning Futures: Planning the Next Century’ by suggesting future health outcomes are and can be secured by creating resilience in current planning policies, and that in order to achieve a resilient set of planning policies, we need to gain a complete evidence-informed understanding of these policies as part of a wider futures-thinking and systems approach.

While planning systems vary from country to country, the focus of this article on England allows a deep dive into one system to identify transferrable international lessons for other comparable plan-led systems. As many local planning systems rely and require production of a spatial plan or strategy, it validates this focus on a statutory instrument as an important public health intervention.

We now have an overwhelming wealth and depth of understanding of the complex relationships that exist between the physical environment and health (Rao et al. 2007; McKinnon et al. 2020; Chang, Green and Petrokofsky 2022). This article puts forward an evidence-based analysis on the function of the statutory local plan as a key lever for expressing spatial planning objectives, including improving health as a social value outcome. It is based on research and practitioner-facing activities of the TRUUD (Tackling the Root causes Upstream of Unhealthy Urban Development) project (Black et al. 2022), to better understand and apply actions on systems of governance further upstream in the urban development process – such as the local plan, to improve population health.

This research frames the local plan, produced by local planning authorities (LPA) as a legal determinant of health and a public health intervention in spatial planning (Gostin et al. 2019; Montel 2023). It recognises the land use policies created through the local plan can help realise health benefits associated with the quality of urban development (Rao et al. 2007; Public Health England 2021), and demonstrates why research recommendations have suggested that health and well-being is central to urban planning policies and practice, particularly when addressing outcomes such as respiratory health impact of air pollution (Chang 2018; McKinnon et al. 2020; Nieuwenhuijsen et al. 2023). Doing so can help drive contemporary objectives of environmental sustainability, social value, health and wellbeing, and inequalities.

Comparative research of local spatial plans is currently limited in the UK and internationally, particularly when focused on health and wellbeing, and comprehensiveness of coverage across all the public bodies in one country. That is why this research adds value because of the completeness of coverage of local plans from LPAs. But there has been some notable contemporary research worth highlighting which helps frame and complement this research, and when undertaking further analysis, provide a reference point for corroboration and challenge. In the UK this includes a health review of all Local Plans in both England (n = 322) and Wales (n = 22) by the Town and Country Planning Association (TCPA) (Chang 2019); another TCPA review of only London boroughs’ (n = 35) planning policies on poverty and inequality (Town and Country Planning Association and Trust for London 2019); and more recently a TRUUD project health review of a seven local plans in England (Callway et al. 2023). Internationally, researchers have been keen to review urban planning policies to determine the comparability of approaches to support health. For example, a global health and sustainability review of cities (n = 25) from high to low income countries was published in The Lancet (Lowe et al. 2022). The above research suggested there is limited presence of health requirements in local city-level planning policies. This can undermine the quality of urban development proposals, which in turn harms efforts in population health improvement through policies set out in statutory spatial plans.

The discussion in this paper is based on the results of a comprehensive policy review of statutory spatial plans, the local plan, created by LPAs in England. The article provides the results of the local plan review through content analysis and combining with a narrative review to begin to address the hypothesis of the statutory local plan as a public health intervention. This article takes an integrative approach by presenting a discussion based on an analysis of how current local plans can contribute towards healthy urban development.

### Status of the Local Statutory Plan in England

The local plan has long been used by planning and housing authorities to exercise control over the location and form of urban renewal. The origin of the planning system in nineteenth Century industrial settings was to specifically manage environmental health issues from acute situations such as tackling the population health effects of water and air pollution. The Housing, Town Planning, &c. Act 1919 requires local authorities to prepare a town planning scheme in respect of all land within the borough or urban district. In the 1920s, local plan preparation and content were subject to guidance under the responsibility of the UK Government Ministry of Health, while in the 1940s, the creation of the foundations of the planning system and the national health service was overseen by a joint minister for housing and health (Hebbert 1999). The planning system’s historic relationship to health has been direct and intentional.

Currently such responsibility for national guidance has moved to the UK Government Department for Levelling Up, Housing and Communities (DLUHC) while responsibility for producing local plans remain with LPAs. The origin of the planning system has evolved into the modern system as a strategic planning process and development management lever of land uses within the built and natural environments in which people live, work and play (Ministry of Housing, Communities & Local Government 2015; Raynsford Review 2018). As such the scope of the planning system reach across the environment is now expansive, and is set out in the National Planning Policy Framework (NPPF) (Department for Levelling Up, Housing and Communities 2021). This is positive in terms of reflecting the issues covered by the wider determinants of health within the scope of spatial planning (Public Health England 2017b, 2021), but also a threat because of the potential that if not reflected in local plans, the issues may not be addressed in planning decisions (O’Malley et al. 2021; Chang, Green and Petrokofsky 2022).

The status of the local plan and the ‘plan-led’ system in England is established in planning legislation by the Town and Country Planning Act 1990 (with various subsequent amendments) which states that in determining applications for planning permission LPAs should have regard to the provisions of the development plan or the local plan). The continuing emphasis on the primacy of the local plan can be demonstrated by government efforts to reform the local plan system (Local Plans Expert Group 2016; Ministry of Housing, Communities and Local Government 2020) and ministerial directions (the highest level of political articulation of policy) to LPAs. In 2021, the then-Minister of State for Housing, Rt Hon Christopher Pincher MP, published a written statement to commit LPAs to not delay plan-making (Rt Hon Christopher Pincher MP 2021). At the time the Minister reiterated the Government’s commitment from March 2020 to set an ambitious deadline of December 2023 for all authorities to have up-to-date Local Plans in place (Ministry of Housing, Communities and Local Government 2020), though this deadline has not being met due to uncertainty and delays in implementing Planning Reform throughout 2023.

### Research Aims and Objectives

The local plan policy review in this article provides the initial steps in addressing the hypothesis that embedding health intentions into local plans is a prerequisite to effective integration between planning and public health systems, resulting in securing healthy developments. This hypothesis will be tested initially by the results of this review to gain a foundational understanding and identifying whether wider determinants of health indicators are contained in planning policies within each England local planning authority’s statutory plans. The following objectives are expected:

- To determine number of local plans with health policies that are NPPF compliant and address any of the wider determinants of health.
- To understand how these health policies are worded/phrased.
- To have findings in presentable formats that are capable of comparative analysis to discuss and update relevant previous local plan reviews.

There are points at the intersection between the spatial planning system and urban development journey where actions undertaken can lead to certain consequences for population health – physical and mental health and wellbeing, and health inequalities. It is not the intention to undertake another systematic review of literature given the existence of a strong body of academic evidence (including recent ones based on systematic review methods) on the relationship between the built and natural environment and people’s health (Power et al. 2009; House of Lords Select Committee on National Policy for the Built Environment 2016; Commission on Creating Healthy Cities 2022) and the role of local authorities through their planning functions (Local Government Association 2020). Therefore, the premise of the local plans review adopts the research commissioned and published by Public Health England in 2017 as an evidence resource, undertaken by the University of the West of England for the English audience and context (Public Health England 2017b). This research by Bird et al., through an umbrella review method, identifies five themes (including 15 principles and 39 attributes) regarding the built and natural environment for which there is robust path-ways of evidence that they support good population health and the prevention of ill-health (Bird et al. 2018):

1. Neighbourhood design: impact on our day-to-day decisions and therefore have a significant role in shaping our health behaviours,
2. Healthy homes: Living in good quality and affordable housing is associated with numerous positive health outcomes for the general population and those from vulnerable groups,
3. Healthier food and the food environment: limited quality review level evidence on the influence of the food environment on health and wellbeing outcomes,
4. Natural and sustainable environment: contact and exposure to the natural environment has implications for improved health and wellbeing,
5. Transport, including active travel infrastructure and public transport: Active travel can increase physical activity levels and improve physical and mental wellbeing.

## Methods

The review is based on a two-stage protocol (Chang and Carhart 2023) as informed by the knowledge and experience of an expert panel for the purpose of ensuring the robustness and relevance of the research. The panel was made up of an associate director in a private sector planning consultancy, managing director of a healthy building consultancy, head of a planning improvement service, planning for health specialists in local government, a public health lecturer and a systems lecturer. A first meeting of the Panel was held in December 2022 in which the Terms of Reference was discussed and agreed followed by a second meeting in July 2023 to present and discuss the results. No ethics was required after screening by the relevant university ethics committee.

This article focuses on Stage 1 of the protocol, hereafter referred to as the census. The census was a desktop-based survey of each current local plan in England conducted between January and June 2023. The census aims to provide a complete enumeration of the ‘population’ of local plans at a point in time with respect to a set of defined parameters, with the ability to disaggregate results to the nine English geographical regions.

The review framework (Table 1) consists of these 26 health parameters and was compiled to build an evidence-led health picture of each local plan. The parameters covered the following areas:

- achievement of government national planning policy requirements for public health system considerations such as links to statutory local health needs and strategies (parameters 1–3)
- existence of a strategic local spatial objective and policy on health (parameters 4–6)
- existence of local policies on health based on PHE evidence review’s 5 themes (parameters 7–13)
- adoption of healthy planning frameworks and monitoring indicators that can be used as proxies for implementation (parameters 14–15)
- existence of policies on areas of emerging public health interest by the UK government (Department for Levelling Up Housing and Communities 2022; Department of Health and Social Care 2023) such as biological sex or specifically women and girls, mental health, security and suicide prevention considerations (supplementary parameters).

After the initial screening based on an inclusion and exclusion criteria to establish the scope and scale of the review documents, we established a final sample size of LPAs and their local plans. Note that some LPAs were excluded as their local plans were not eligible due to the year of adoption before 2012, and some LPAs have two local plans (containing strategic and detailed policies) which were both eligible for inclusions. These factors resulted in determining a final sample LPA size of 315 from which a total of 346 local plans were reviewed. The review progressed to sourcing each individual eligible local plan document on local authority website as a Portable Document Format (PDF), and to file each document for review.

**Figure 1:**
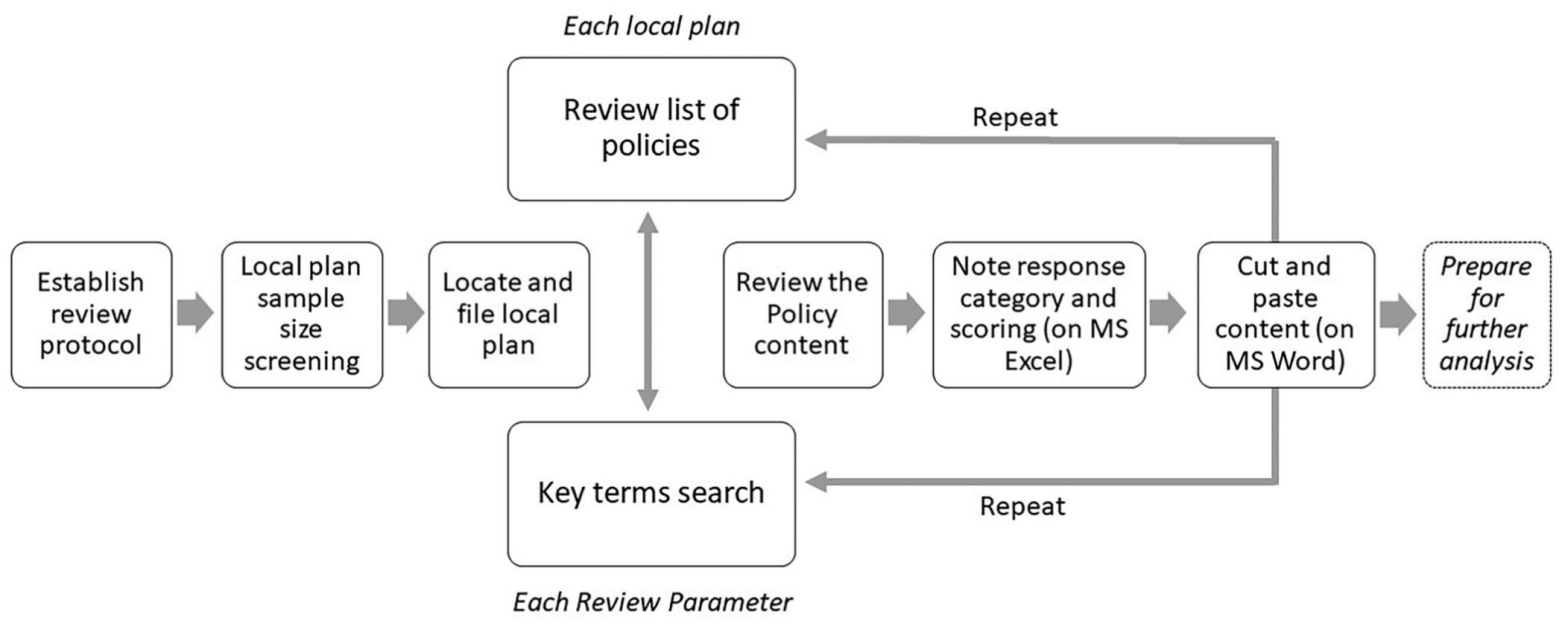
Local Plans census review process.

We undertook the census using Adobe Acrobat according to the following process for each local plan document and each review parameter (Figure 1):

- Review local plan list of policies
- Undertake key terms search
- Go to the Policy and read through the Policy and/or Supporting Text.
- Note response against the Response categories to complete the census (on Excel). For parameters that do not require a binary ‘yes’ or ‘no’ response, the following response categories were applied:
  - Yes (reference to health in policy): where the term ‘health’ appears in the main body of the policy text.
  - Yes (reference in supporting text): where the term ‘health’ appears only in the supporting text to the policy.
  - Yes (but no reference to health): where the term ‘health’ does not appear in both the main body of the policy text and the supporting text.
  - No: where no policy exists.
- Attribute scoring to each response for the purpose of listing review results and ability to conduct quantitative analysis to the responses. Scoring was not used and intended as a means to rank local plans or determine their health impact and effectiveness.
- Cut and paste relevant Policy and/or Supporting Text for further thematic analysis, if selected for the next stage (on MS Word)

To ensure relevant contents are identified and accounted for during the search process, we set out key terms and suggested likely synonyms under each parameter in the review protocol. For example under active travel policies, the following terms were identified within the search parameters – active travel, walking and cycling, and active transport. Further methodological notes and observations were kept under a separate tab in MS Excel, which was the primary mechanism for recording the review results, as a way to reflect and continuously improve consistency during the process.

## Results

We present the census results in Table 2 for England as a whole and for each English region (*n* = 9). We undertook a quantitative content analysis for the census based on scoring the responses recorded for each local plan against the review frame-work. A summative content analysis based on quantitative responses to the census is determined to be the most appropriate approach involving counting and comparisons of the local plans content, followed by the interpretation of the underlying context (Hsieh and Shannon 2005). This is because, without input of contextual factors, any analysis at this early stage of result analysis would be premised on assumptions such as trends and themes which cannot be verified yet.

## Discussion of Results

Legislation requires planning decisions to be made in regard to the local plan. It is known from contemporary research on local plans that they can contain a wealth of insight into spatial planning as a legal determinant of health (McKinnon et al. 2020; Lowe et al. 2022; Callway et al. 2023). They are readily available and accessible public documents which are essential to all users of the planning system in not only determining consent for individual land use applications but also getting a sense of the strategic ambitions of the local authority over the next 15 years of each local plan period.

The research establishes a baseline of understanding into the central mechanisms by which local plans facilitate the building blocks of population health. Without establishing a baseline of current state of planning policy we cannot understand the state of public health spatial planning in practice. With the understanding enabled by the census for England, we can begin to comprehend how and whether the range of wider determinants that are enabled through planning could be made to improve population health for current and future generations. Key themes emerge from the census and further analysis using additional layers of contextual data such as but not limited to local authority type, deprivation, socio-economic status, and political makeup will be undertaken and published in future papers.

### Strategic Recognition of Health

Existence of key and specific health terminology in local plans is essential to ensure decisions can be framed to deliver on outcomes, not least because the time frame of local plans extend 10–15 years into the future. But the absence of a definition of health and wellbeing in the context of planning and explicit direction to include health policies in national government legislation and policy, such as in the NPPF, should be noted (Montel 2023). This is despite the NPPF indicating a social objective of the planning in achieving sustainable development is to support healthy communities with places “that reflect current and future needs and support communities’ health, social and cultural well-being” (Department for Levelling Up, Housing and Communities 2021). Noting a highly centralised nature of the English planning system (Hambleton 2017), a lack of a clear legal and policy narrative from national government can result in diluted application and recognition of planning for health in local policy as reflected in the census results.

**Figure 2:**
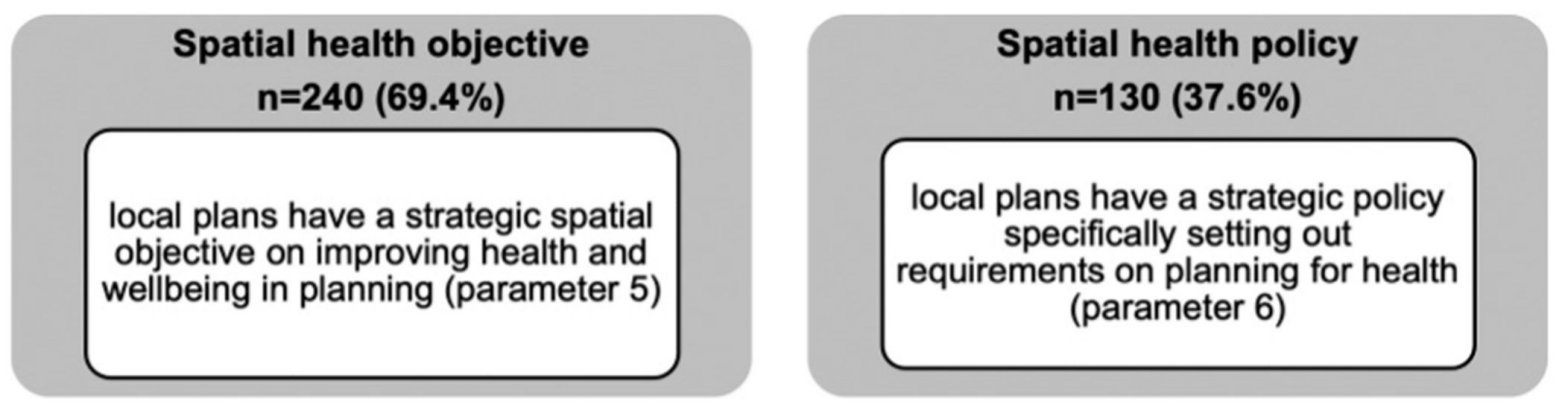
The headline results for strategic health considerations.

Figure 2 shows 240 (69.4%) local plans have a strategic spatial objective on improving health and wellbeing of the population. For example, Herefordshire’s local plan states: “*To improve the health, well-being and quality of life of all residents by ensuring new developments positively contribute towards better access to, provision and use of, improved public open spaces, sport and recreation, education, cultural and health facilities, local food production and ensuring safer communities*”. These spatial objectives are part of the local plan vision statement and reflect wider corporate planning for the authority which will usually be all-encompassing and therefore include improving health as an objective.

Of those local plans with a spatial objective on health, only 111 (46.3%) go on to develop a specific spatial health policy (a total of 130 local plans has a health policy). This may suggest while LPAs have a high-level recognition of health as an objective, most have either not recognised the need to develop a specific policy or lack understanding of what a health policy could look like. Only further analysis through mixed methods including direct engagement with LPAs will determine the exact reasoning. To supplement our understanding to determine whether health is recognised in local plans, a word search of ‘health’ and ‘wellbeing’ found that the national average in each local plan is 93 and 19, respectively, with ‘health’ ranging from 65 to 154, and ‘wellbeing ranging from 12 to 27 mentions. This observation is supported by results of the word search of ‘mental health’ which yielded on average only 3 mentions in a local plan though it should be noted that limitation of the census approach which does not further disaggregate and refine information about specific mental health outcomes such as dementia or prevalence of depression. In addition, the census found some LPAs have adopted separate planning guidance known as supplementary planning documents (SPD) on planning for health (n = 21).

### Is Health Reflected in Some or All Policies?

**Figure 3:**
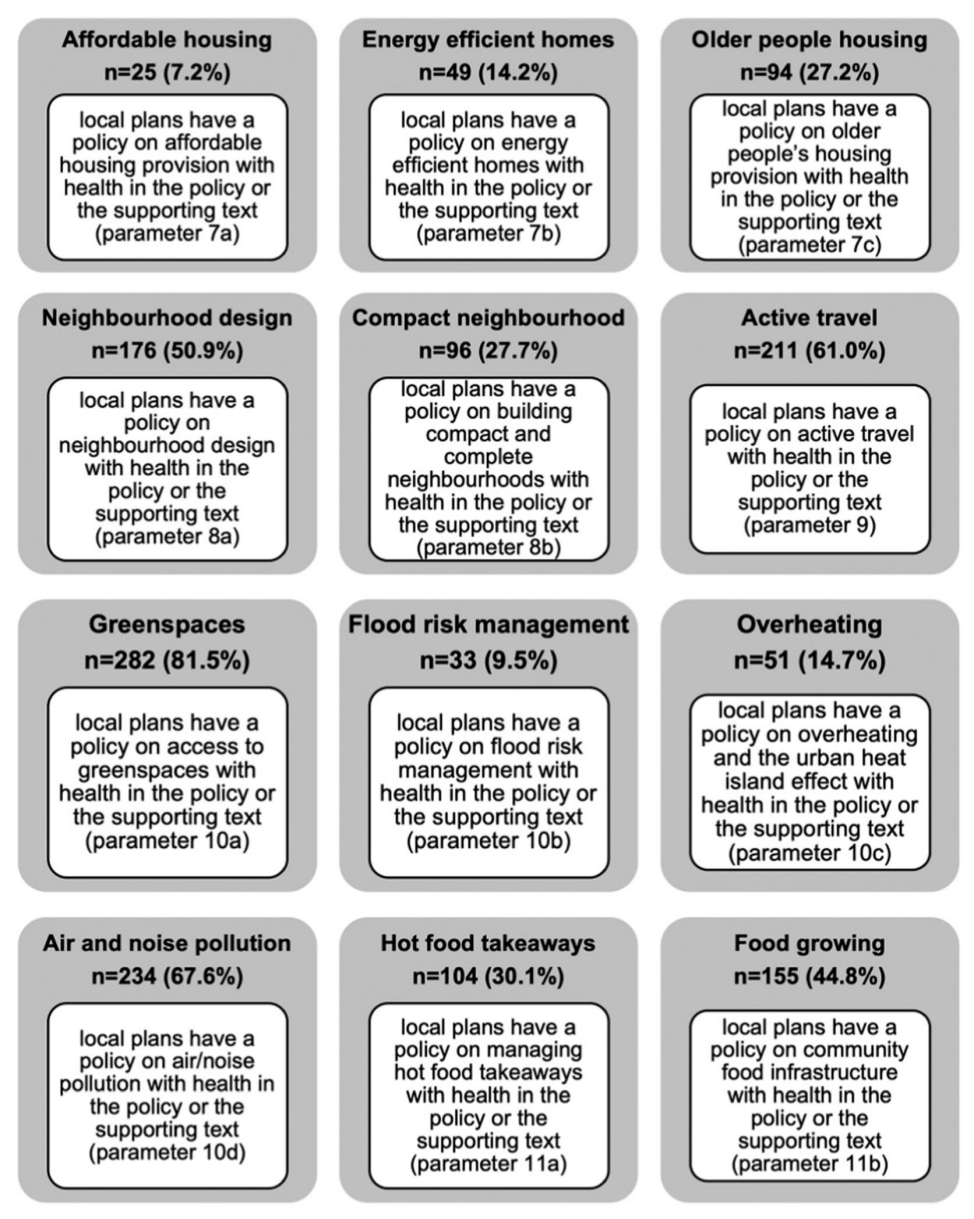
Headline results showing number of local plans responses according to review parameters.

**Figure 4:**
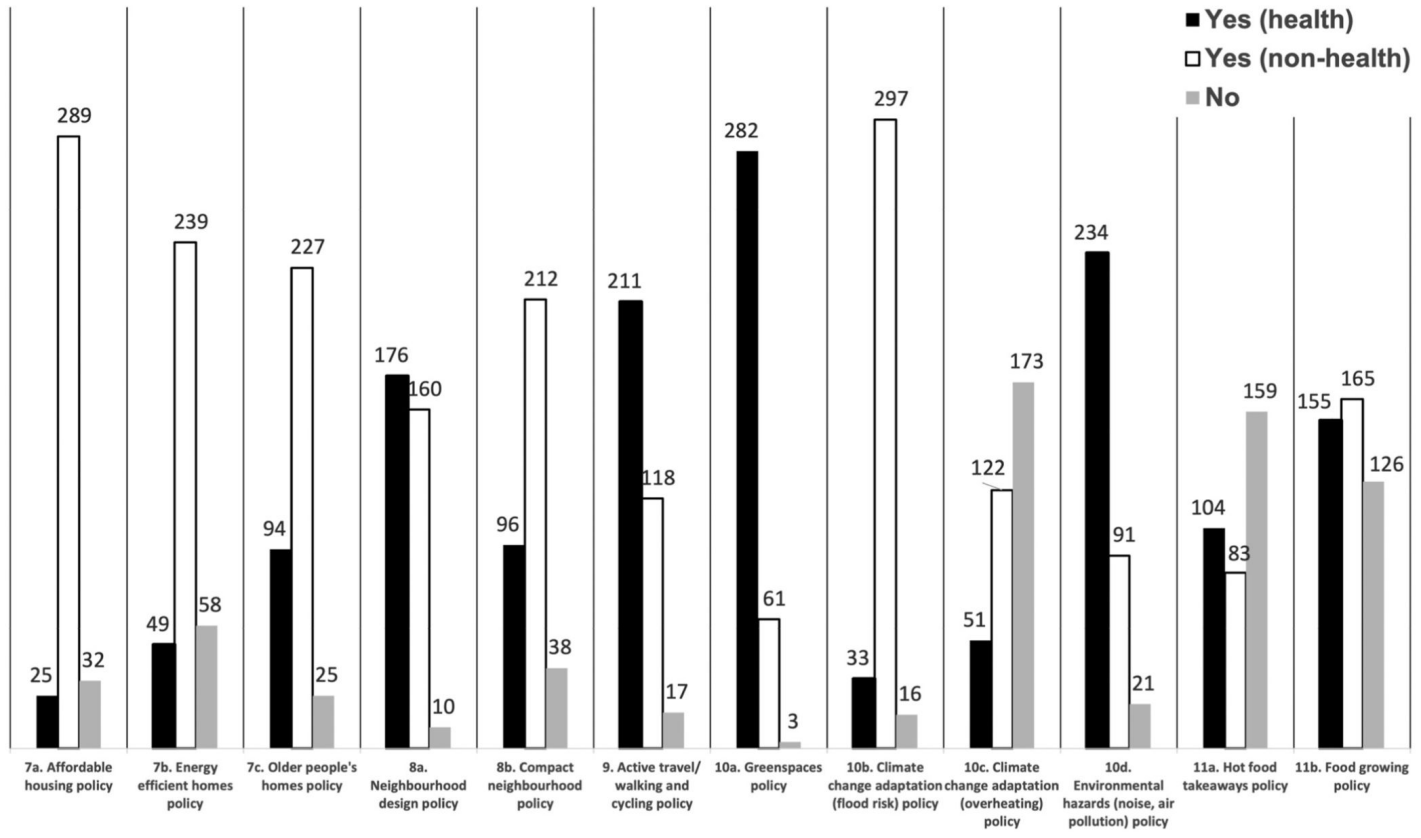
Coverage of local plan policies against health-evidenced parameters 7–11.

There is an increasing interest on a health in all policies approach to work across those systems that can influence the health determinants such as the planning system to impact on health inequalities and improve wellbeing (Greszczuk 2019; Chang, Green and Petrokofsky 2022). In this regard, we should expect to see greater spread of health references in policies across the census parameters 7 to 11 from housing to the natural environment. The census indicates limited coverage across local plans, which suggests health is not currently reflected across all policies as shown in Figure 3 and visualised in the summary graph (Figure 4), a finding supported in a health study of 25 global cities’ policy frameworks that only one city scored highly for policy coverage across the health indicator categories (Lowe et al. 2022).

This can suggest that planners’ understanding is limited to some established policy areas such as green spaces and air/noise pollution because of the established and well-known evidence and knowledge in these areas. But health has not been referenced in connection with other prominent areas such as policies on affordable housing, climate change and the food environment. While this does not suggest that positive health outcomes may not materialise, but it poses challenges as to whether promoting the approach to embed health across multiple policy areas is working in practice. It begs the question whether a central coordinating health policy, as many councils already have (see earlier results in Figure 2), may be more effective than dispersing health across some selective policies.

Another explanation is the presence of absence of requirements set out by national government in the National Planning Policy Framework (NPPF). For example results for parameter 10c on overheating and resilience to urban heat island effect show limited reference to health in this policy or indeed presence of any policy as 173 (50.0%) of local plans do not have an overheating policy. This can be partly explained because consideration of climate change and overheating was only introduced into NPPF in 2018 which states that “plans should take a proactive approach to mitigating and adapting to climate change, taking into account the long-term implications for flood risk, coastal change, water supply, biodiversity and landscapes, and the risk of overheating from rising temperatures” (Department for Levelling Up, Housing and Communities 2021). Further longitudinal analysis to overlay policy and other directions from government can yield interesting insight on the relationship between national and local policy.

### Public Health and Planning Systems Alignment

Reviewing how the public health and planning systems align is important to understand whether identified health needs and priorities of the statutory local public health functions are being systematically considered in the local plan. There is a policy basis for this approach as the NPPF requires planning policies to “*enable and support healthy lifestyles, especially where this would address identified local health and well-being needs*” which effectively refers to the statutory joint strategic needs assessment (JSNA) undertaken by the local public health department, and to “*take into account and support the delivery of local strategies to improve health, social and cultural well-being for all sections of the community*” which effectively refers to the joint health and wellbeing strategy (JHWS) also undertaken by the local public health department. These two documents are key mechanisms for directing local health systems to consider the direct and indirect impacts of their functions on health. It is therefore important that the built and natural environment, as building blocks of health, is adequately recognised within them as suggested in statutory government guidance (Department of Health 2013).

**Figure 5:**
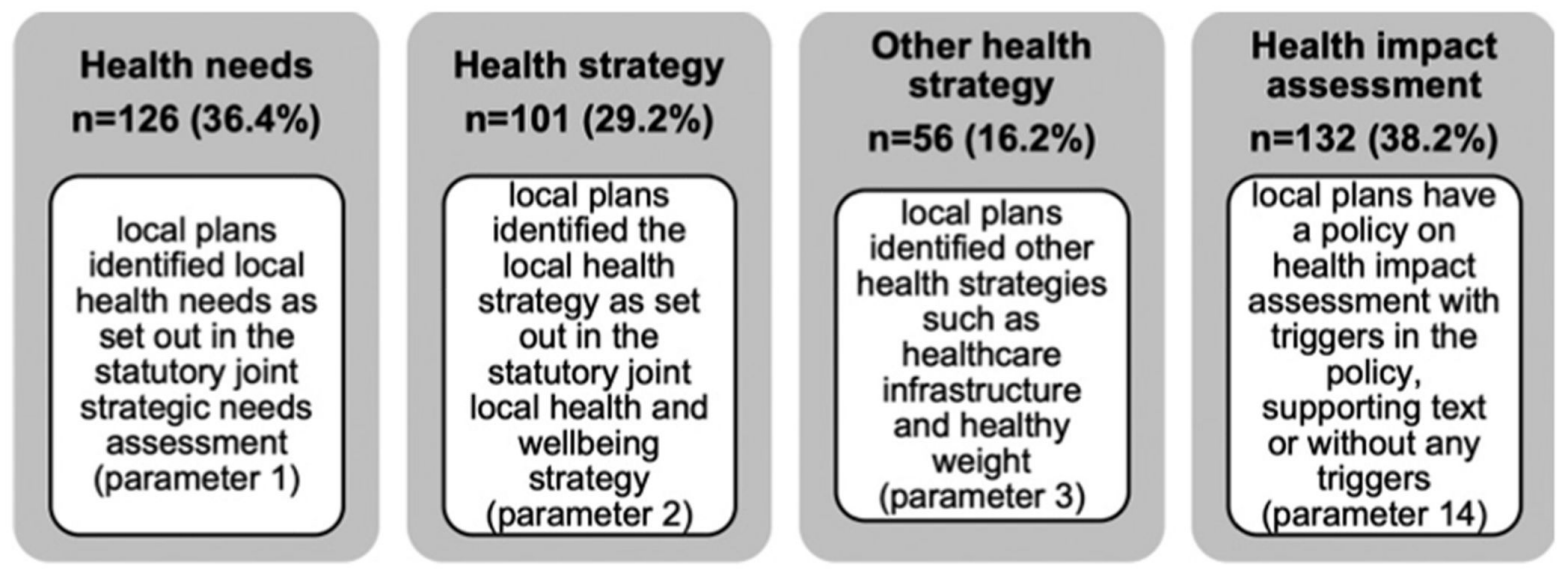
Headline results for health and planning system links.

Figure 5 shows the census results. Previously the TCPA review in 2019 found 27% of local plans refer to the JSNA and 23% refer to the JHWS (Chang 2019). This census in 2023 found slight improvements with 36.4% of local plans referring to the JSNA and 29.2% referring to the JHWS (see Table 3). This suggests either LPAs are still largely unaware of the existence and relevance of public health strategies in spatial planning (as supported by findings of the limited sample review by Callway, see (Callway et al. 2023), that such strategies do not adequately reflect the range of wider determinants of health or have not been created to be applied or implemented through the planning process. One explanation could be due to the institutional discrepancy that exists in two-tier administrative areas with public health functions at the upper tier county council while planning functions remain in the lower-tier district councils (Local Government Association and District Councils Network 2019). Nevertheless, further research will be required but the starting point would be to recognise from a national survey in 2021 that found the majority of the local public health system does have an understanding of tackling the wider determinants of health and wellbeing to a great or moderate extent (Local Government Association n.d.).

However public policy systems such as planning and public health will not stay static and will either evolve or revolutionised depending on a combination of political, societal and fiscal drivers as evidence in post-war planning in the UK (Raynsford Review 2018). So there is a danger that static policy documents such as the local plan can become out of date or invalidated as systems and legislative requirements change. This is one risk that needs to be factored into the day-to-day decisions by LPAs but the central premise still stands that future systems, decisions and practices will be ill-prepared if policies are not put in place in the present time to be resilient to the challenges that will surely arise.

### Geographical Disparity and Clustering

There has been an assumption by planning commentators about the difference in performance between regions such as between London and the South East of England as compared to those in the North East and North West of England (Rowthorn 2010; Turner et al. 2023). For example socio-economic and health data does show a north-south divide where income disparities across the English regions are linked to several health conditions such as obesity and depression (Baker 2019; Whitty 2020) so addressing these disparities has been a main driver force behind the Government’s levelling up agenda (HM Government 2022). It should be noted that such disparity also exists within an area between the most and least affluent neighbourhoods but the national scope of the census, means our focus at this stage is more feasible on the macro than micro scales.

The census paints a spatial picture of policy across the nine English regions and across the review parameters. For example, policies on parameters that contribute to health outcomes such as access to greenspaces for health indicate a higher level in South East of England local plans where 21.1% of local plans include references as compared to the North East local plans at 15.4%. However, there appears not to be a consistent pattern observed in policy disparities. For example, a policy relating to managing less healthy hot food takeaways, only 2.6% of local plans in the South East have one while North East has a higher proportion at 46.2%. This poses the question whether any specific disparity is influenced by policy or is the policy in response to an attempt to address a regional or local health disparity. Further research using longitudinal methods may be needed to better understand such underlying trends and the evolution of the policy geographies of health.

There is also an observed clustering effect where LPAs in geographical proximity, usually in a sub-region, display similar or aligned policies. For example, aggregated census findings from LPAs in defined greater-than-local geographies found those in, for example, Greater London (64.9%), Greater Manchester (50.0%) and Liverpool City Region (50.0%) all have a policy on overheating and UHI (parameter 10c). In part this observation is expected given their highly urbanised setting and likelihood of experiencing hotter temperatures than in more rural geographical settings such as the counties of North Yorkshire (28.6%), Hampshire (23.1%) and Worcestershire (16.7%). But it is unclear whether this clustering effect is intentional or coincidental. The Department for Environment, Food & Rural Affairs (Defra) Rural-Urban Classification for England identifies six categories local authorities as rural or urban based on the percentage of their resident population in rural or urbanised settings (Bibby and Brindley 2016). When analysing the urban heat island (UHI)/overheating parameter where only eight LPAs have a local plan policy on overheating with health, the results show these LPAs are predominantly urban with only one in the predominantly rural category. When overlaying this against regions, London boroughs which are all categorised as predominantly urban have the largest proportion with a policy on overheating (64.9%) though none reference health. The results corroborate with the general understanding of UHI of more urbanised areas with elevated temperatures when compared to rural surroundings (Mentaschi et al. 2022).

Results can also analyse the multiplicity of relationships between existence of policies and local authority characteristics to better understand whether certain local authority types are more likely to have certain policies. An example is analysing the parameter on hot food takeaways against density of take-away outlets from Public Health England statistics by local authority in England (Public Health England 2017a). The review results show that ten LPAs with the least density of hot food takeaways do not have a policy while out of the ten LPAs with the most density of takeaways, which also link to levels of deprivation, four (40%) of them do have a policy. The results corroborate with strengthening research demonstrating the relationship between exposure to and density of take-aways, with deprivation and obesity prevalence (Sawyer et al. 2021).

### Monitoring and Review

The Town and Country Planning (Local Planning) (England) Regulations 2012 requires LPAs to publish information annually that shows progress and implementation or lack thereof of local plan policies. There are no specific requirements about what indicators to adopt so it is up to each LPA’s discretion about what to monitor. The results are reported through the Authority Monitoring Report (AMR) which is a publicly accessible document. During the census, the Monitoring Framework contained in each local plan, usually presented in the appendix, was reviewed to identify existence of any indicators against the census parameters.

Figure 5 shows the existence of indicators to monitor the implementation and effectiveness of planning policies vary across the set of review parameters. This mixed picture of monitoring frameworks in local plans may be explained by the lack of national requirement and direction on monitoring, beginning with the abolition of the local government National Performance Framework set of 198 different performance indicators on areas including on health and wellbeing and environmental sustainability from April 2011 (Lowndes and Pratchett 2012).

**Figure 6:**
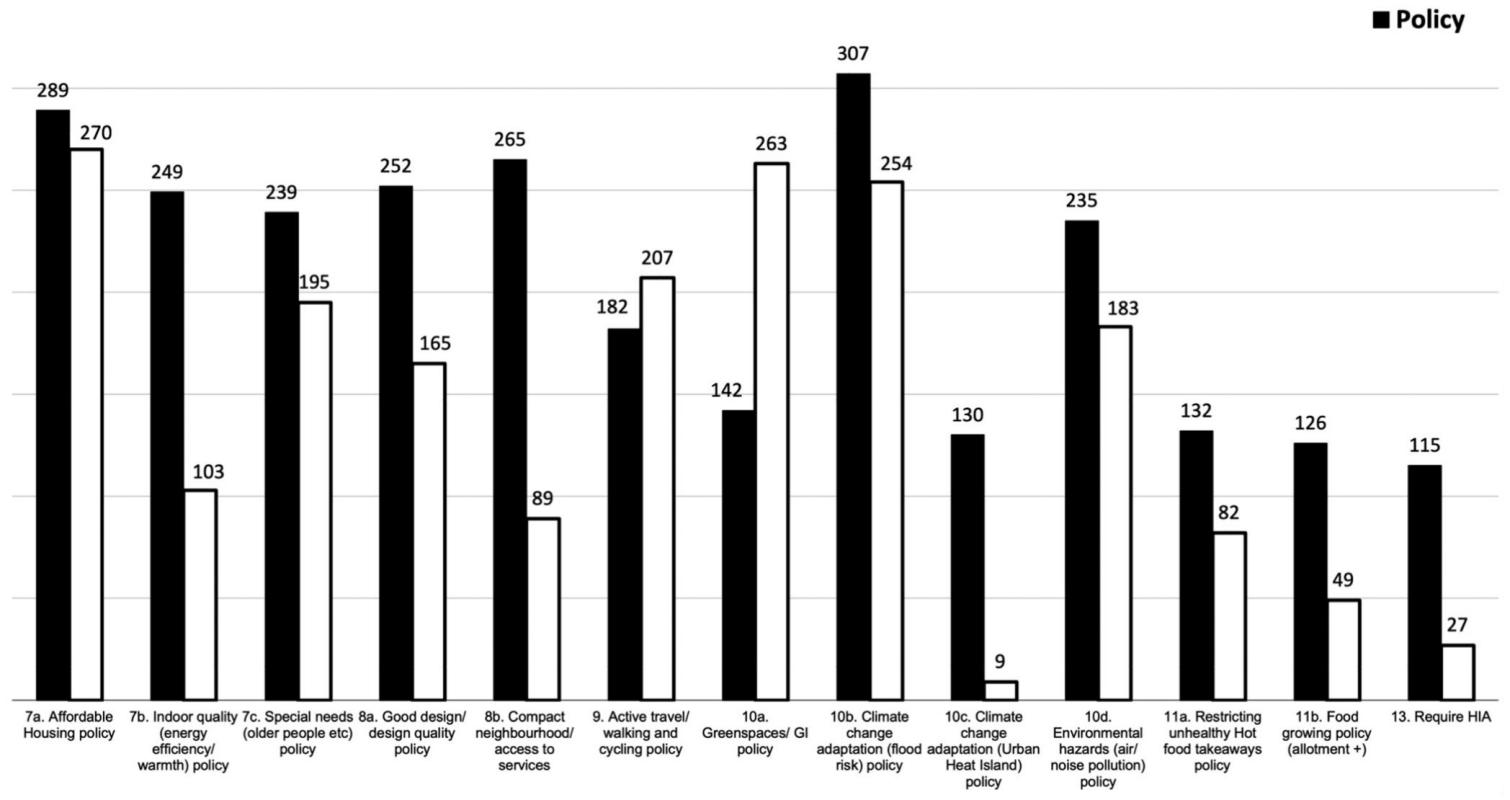
Headline results for indicators in local plan monitoring frameworks.

Figure 6 contrasts the existence of indicators against the existence of the policy. The overlaying of these two elements shows an inconclusive picture of relationship between a local plan containing a policy and existence of an indicator to monitor its implementation. The numbers and nature of indicators will be subject to further analysis but preliminary observation of what these indicators are suggests policies that can be monitoring through direct and quantitative measures are likely to be accompanied by an indicator, for example those relating to the number of affordable housing units delivered in any given year, number of homes built on flood risk areas or the area of greenspaces or cycle lanes built. Figure 5 also provides insight into an anomaly where local plans have indicators on issues in where no related policies exist, such as for active travel.

This finding of a disconnect between policies and monitoring indicators poses challenges on two grounds. Firstly, the lack of and incomplete coverage of monitoring indicators linked to the census parameters may present an obstacle for LPAs to ‘benchmark’, project into the future and better understand the ongoing health impact (positive or negative) of their policies (Giles-Corti et al. 2022). While indicators to monitor affordable housing delivery have been identified in most local plans, those monitoring the use of Health Impact Assessments (HIA) and the food environments are few, even when LPAs have a policy in place. When time comes to review the local plan, usually every five years, there would not be the readily available monitoring data to identify key opportunities to improve or adapt policies.

Secondly, the paucity of monitoring indicators may indicate a lack of understanding or clarity from LPAs of how their future vision, strategic spatial objective on health and outcomes can and should be realised through introducing policies, and through implementation by developers (Callway et al. 2023). For example, not all of the 132 LPAs with a policy to manage over-concentration of hot food takeaways (HFTA) have a monitoring indicator to record the annual number of HFTA applications received and determined. That means they would not be able to determine the location and scale of health impact the policy may achieve, such as in neighbourhoods with high obesity prevalence. Similarly, only 27 of the 132 LPAs with an HIA requirement have a monitoring indicator to record the annual number of HIAs received or reviewed as part of planning applications, which means in future plan reviews they would not be able to determine whether the policy had been achieved or effective.

### Limitations

The census review framework was developed with the primary aim to gather high level response about the absence and/or presence of key terms relating to the wider determinants of health. Though it was not the intention to search for the presence of entirety of medical conditions such as cardio-vascular or respiratory health, as it was assumed that such terms would be associated with the presence of the term ‘health’. The main methods of gathering these responses combined identifying relevant policies followed by key term search function and by reading through these policies. While the review protocol identified a risk with these methods of missing relevant policies given the size of each local plan document and dependency on the knowledge of the individual researcher, any impact on methodological robustness was moderated by peer review of the protocol by the expert panel followed by a test and trial phase undertaken during January 2023 on two sample local plans before commencing the full review.

While a review carried out by one researcher may entrench any inherent bias, the risk was mitigated by the potential variation in knowledge and interpretation of planning terms that may arise when a review is carried out by a team of researchers. One lesson learnt by undertaking a large-scale analysis of documentation such as the local plans census was the need for terms and synonyms are clearly defined and ensure the terms are relevant to the context in which they would be commonly used. Addressing this would ensure the review framework reflected established practice, but also that it can a useful tool when seeking to apply it more internationally, by more than one researcher and by researchers with potential diverse and limited planning knowledge.

## Conclusion and Implications for Research, Policy and Practice

Planning for health does not happen by default and the population’s current health status can be attributed policy actions taken many years ago (Chang, Green and Petrokofsky 2022). This perspective on the need for spatial planning frameworks to be explicit about the articulation of health objectives, needs, priorities and outcomes had been highlighted as an area for planners and local governments to address. Notably by the UK Parliament’s health select committee in its various enquiries into childhood obesity which issued the specific recommendation to Government that “health should be included as a material planning consideration” in 2015 (House of Commons Health Committee 2015) then again in 2017 (House of Commons Health Committee 2017). The National Planning Policy Framework requires planning policies to take into account identified local health needs and support delivery of health and wellbeing strategies. As such this research is timely to establish a baseline of current local planning policy and health understanding.

Further detailed qualitative analysis will be undertaken as part of the next stage of the local plans review to better understand the complex and hidden relationships behind the census findings. Such analysis will review the policy wording identified from the census of a selected sample of local plans. The analysis will follow the thematic analysis process (Braun and Clarke 2022) and supported by the use of software such as NVivo and geospatial mapping techniques. Further analysis will also review and overlay selected contextual information such as local authority type, local plan adoption date, population and housing growth, public health outcomes data, well-being and deprivation indices, and presented in mapping and other appropriate visual formats. Findings from such further analysis will be published in future articles.

From the census, we can observe an increasing awareness of LPAs in England how the spatial planning system can influence health and wellbeing and willingness among planning professionals to integrate principles of healthy placemaking in their policies and decisions (Ross and Chang 2012; Royal Town Planning Institute 2020). The results of this novel local plans review and the census has yielded useful insights into the extent that authorities are integrating public health into spatial planning in England especially to compare with the state of the union report by the Town and Country Planning Association in 2019. The results are important to note because the local plans reviewed are final, adopted and published by LPAs, and therefore present the preferred option for those areas when planning and implementing urban development. It would be interesting to review and analyse whether previously discounted options in earlier stages of local plan-making, in following good practices in scenario planning (Schoemaker 2004; Chakraborty and McMillan 2015), could be conducive to delivering different levels of health outcomes.

When aligning this observation with comparative international research, there is an overarching consensus on the need for health and wellbeing to feature more prominently in future urban planning policies and practices (United Nations Human Settlements Programme 2022). A number of comparative UK-based and international health reviews of planning policies were highlighted earlier in the paper. While their methodological approaches and health review frameworks employed have varied, their results and conclusions have been consistent in recommending that more needs to be done to build capacity “for health-enhancing city planning policy and governance” (Lowe et al. 2022). The results presented by the census in this paper support this recommendation and provides a robust foundation from which to conduct further analysis and primary research to address the issues at the core of the local planning system in England.

The census results highlight several local policy gaps such as the absence of overheating and the food environment policies across all local plans and addressing the health impact of housing policies, as well as areas for potential research focus on specific parameters relating to particular health challenges in England. In this census, we found that around one third of local plans in England align identified local health needs and priorities into policies, which is a slight improvement on the TCPA review in 2019 but the majority of LPAs are still not in full compliance with national planning policy requirements. Echoing the TCPA report which observed, “t*here is still a significant lack of alignment between the planning and health systems, but as many new Local Plans/Local Development Plans are being developed or reviewed, the state of planning for health policy can only improve*” (Chang 2019).

One explanation for the limited local plans adoption progress can be placed on the national planning reform programme. A backdrop of new proposals to reform the planning system in England by the UK Government since 2010 have had and will continue to have a profound consequential effect on the adoption, quality and effectiveness of LPAs to deliver outcomes through their local plans, such as relating to housing and public health. Other impacts have been identified by a range of planning commentators, experts and parliamentarians on the purpose and mechanics of the planning system, the morale of the planning profession and the resourcing of planning administrators in local authorities (Raynsford Review 2018; Clifford 2022; House of Lords Built Environment Committee 2022). These impacts have been compounded by significant reduction in local government resourcing reported to be 43% between 2009/10 and 2020/21 with the North East region’s reduction falling the most at 62% (Steele and Bauer 2022). Callway et al. had suggested further analysis in relation to local government resourcing impact may indicate whether LPA resourcing has an impact on the implementation effect of local spatial planning policies on health (Callway et al. 2023).

While planning futures will not and cannot be an exact science, this paper presented the results of a review based on established literature of evidence that highlight indicators of physical and mental health and wellbeing or widely known as the wider or social determinants of health. These indicators include housing, active travel, access to green spaces and the food environment. But it should be noted that, just as the nature of health challenges we face in the twenty-first century are different to those challenges in the nineteenth century, our public policy systems need to be responsive to current needs while being resilient to different health challenges and opportunities in prevention advancement we will surely come across in the future.

In summary the ongoing disparity and variation in policy geography of local plans for health should be a concern to policy makers who, through the Levelling-up and Regeneration Act 2023 have pledged to levelling up across the United Kingdom by ending the geographical inequalities so that people everywhere can be living longer and more fulfilling lives, and benefitting from rises in living standards and well-being (Department for Levelling Up, Housing and Communities 2020). Further research and analysis of the local plan census to better understand this geographical policy variation and effect of policy clustering would be useful. Public health leaders recognise that preventing poor health should be at the heart of planning and that a responsive and resilient planning system can deliver positive health outcomes associated with the wider determinants of health (Hubber 2023). This census provides a useful set of results to inform national and local policy improvements, and establish an essential baseline from which to create, improve and refine planning policies that can benefit current and future population health and wellbeing.

## Figures and Tables

**Table 1:** Local Plans Review Framework for the Census.

#	Main review parameters	Response	
1	1. Local Plan reference the statutory Joint Strategic Needs Assessment	Yes	No
2	2. Local Plan reference the statutory Joint Health and Wellbeing Strategy	Yes	No
3	3. Local Plan reference to other health-related strategy (for example healthcare facilities, obesity or healthy weight)	Yes	No
4	4. Local Plan subject to a standalone health impact assessment	Yes	No
5	5. Local Plan strategic objective for health	Yes	No
6	6. Local Plan strategic policy on health	Yes	No
7	7. Local Plan policy on Housing:	Yes (reference to health in policy) Yes (reference in support text) Yes (but no reference to health) No	
	a) affordable housing		
8	b) energy efficient homes		
9	c) housing to meet older people and other special needs		
10	8. Local Plan policy on Neighbourhood Design:		
	a) design quality		
11	b) compact neighbourhood/access to services		
12	9. Local Plan policy on transport: active travel		
13	10. Local Plan policy on Natural and Sustainable Environment:		
	a) green spaces		
14	b) flood risk management		
15	c) urban heat island and overheating		
16	d) air and noise pollution		
17	11. Local Plan policy on Food Environment:		
	a) hot food takeaways		
18	b) food growing		
19	12. Local Plan policy on social value and use of developer contributions on social infrastructure		
20	13. Local Plan policy on use of key national healthy planning frameworks or accreditation schemes		
21	14. Local Plan policy on use of health impact assessment in planning applications	Yes (with trigger) Yes (no trigger) Yes (in support text) No	
22	15. Local Plan monitoring indicators for Parameters 7–21	Yes	No
23	16. Supplementary parameters:	Yes	No
	a) Women and girls recognition in planning		
24	b) mental wellbeing recognition in planning		
25	c) security and crime prevention		
26	d) suicide prevention in planning		

**Table 2:** Overview of Census of Local Plans for England Total and Individual Regions.

Review parameter	Response	England	London	South East	South West	East of England	West Midlands	East Midlands	Yorkshire & Humber	North East	North West
Total local plans reviewed		346	37	76	40	47	27	43	22	13	41
		*Percentage of local plans reviewed*									
1. Link to Joint	Yes	36.4%	62.2%	17.1%	22.5%	48.9%	29.6%	41.9%	36.4%	46.2%	46.3%
Strategic Needs Assessment	No	63.6%	37.8%	82.9%	77.5%	51.1%	70.4%	58.1%	63.6%	53.8%	53.7%
2. Link to Joint	Yes	29.2%	45.9%	22.4%	12.5%	34.0%	33.3%	34.9%	45.5%	7.7%	26.8%
Health and Wellbeing Strategy	No	70.8%	54.1%	77.6%	87.5%	66.0%	66.7%	65.1%	54.5%	92.3%	73.2%
3. Link to other health strategy	Yes No	16.2% 83.8%	32.4% 67.6%	14.5% 85.5%	5.0% 95.0%	25.5% 74.5%	11.1% 88.9%	9.3% 90.7%	18.2% 81.8%	23.1% 76.9%	12.2% 87.8%
4. Subject to health impact assessment	Yes No	19.4% 80.6%	32.4% 67.6%	10.5% 89.5%	20.0% 80.0%	2.1% 97.9%	11.1% 88.9%	14.0% 86.0%	36.4% 63.6%	61.5% 38.5%	29.3% 70.7%
5. Strategic (spatial) objective for health	Yes No	69.4% 30.6%	73.0% 27.0%	55.3% 44.7%	70.0% 30.0%	74.5% 25.5%	77.8% 22.2%	81.4% 18.6%	63.6% 36.4%	100.0% 0.0%	68.3% 31.7%
6. Strategic policy on health	Yes No	37.6% 62.4%	54.1% 45.9%	26.3% 73.7%	27.5% 72.5%	31.9% 68.1%	44.4% 55.6%	44.2% 55.8%	27.3% 72.7%	61.5% 38.5%	43.9% 56.1%
7a). Affordable housing	Yes (health in policy)	0.0%	0.0%	0.0%	0.0%	0.0%	0.0%	0.0%	0.0%	0.0%	0.0%
	Yes (no health)	83.5%	83.8%	80.3%	75.0%	85.1%	96.3%	79.1%	81.8%	76.9%	85.4%
	Yes (in support text)	7.2%	16.2%	6.6%	15.0%	2.1%	0.0%	7.0%	9.1%	15.4%	4.9%
	No	9.2%	0.0%	13.2%	10.0%	12.8%	3.7%	14.0%	4.5%	7.7%	9.8%
7b) Energy efficient homes	Yes (health in policy)	2.9%	0.0%	2.6%	0.0%	2.1%	0.0%	4.7%	9.1%	15.4%	2.4%
	Yes (no health)	69.1%	83.8%	64.5%	65.0%	74.5%	70.4%	55.8%	68.2%	46.2%	68.3%
	Yes (in support text)	11.3%	16.2%	9.2%	7.5%	12.8%	7.4%	11.6%	13.6%	23.1%	12.2%
	No	16.8%	0.0%	23.7%	27.5%	10.6%	22.2%	27.9%	4.5%	15.4%	17.1%
7c) Housing to meet older people and other needs	Yes (health in policy)	3.5%	2.7%	3.9%	5.0%	2.1%	3.7%	2.3%	4.5%	0.0%	4.9%
	Yes (no health)	65.6%	70.3%	60.5%	55.0%	63.8%	81.5%	74.4%	68.2%	76.9%	61.0%
	Yes (in support text)	23.7%	24.3%	26.3%	25.0%	21.3%	11.1%	20.9%	22.7%	23.1%	24.4%
	No	7.2%	2.7%	9.2%	15.0%	12.8%	3.7%	2.3%	4.5%	0.0%	9.8%
8a) Design quality	Yes (health in policy)	26.6%	32.4%	15.8%	17.5%	34.0%	25.9%	30.2%	50.0%	38.5%	24.4%
	Yes (no health)	46.2%	37.8%	55.3%	52.5%	53.2%	59.3%	30.2%	22.7%	38.5%	48.8%
	Yes (in support text)	24.3%	29.7%	22.4%	25.0%	12.8%	14.8%	27.9%	27.3%	23.1%	24.4%
	No	2.9%	0.0%	6.6%	5.0%	0.0%	0.0%	11.6%	0.0%	0.0%	2.4%
8b) Compact and complete neighbourhoods	Yes (health in policy)	15.3%	21.6%	9.2%	12.5%	12.8%	11.1%	14.0%	9.1%	38.5%	22.0%
	Yes (no health)	61.3%	59.5%	56.6%	65.0%	66.0%	70.4%	62.8%	68.2%	30.8%	53.7%
	Yes (in support text)	12.4%	16.2%	14.5%	15.0%	10.6%	7.4%	11.6%	13.6%	23.1%	9.8%
	No	11.0%	2.7%	22.4%	7.5%	10.6%	11.1%	11.6%	4.5%	7.7%	14.6%
9. Active travel	Yes (health in policy)	18.5%	35.1%	11.8%	10.0%	17.0%	11.1%	30.2%	27.3%	15.4%	14.6%
	Yes (no health)	34.1%	21.6%	39.5%	40.0%	31.9%	37.0%	23.3%	31.8%	38.5%	34.1%
	Yes (in support text)	42.5%	37.8%	43.4%	40.0%	38.3%	44.4%	34.9%	40.9%	46.2%	48.8%
	No	4.9%	5.4%	5.3%	10.0%	12.8%	7.4%	11.6%	0.0%	0.0%	2.4%
10a) Greenspaces	Yes (health in policy)	23.4%	21.6%	21.1%	17.5%	25.5%	29.6%	30.2%	40.9%	15.4%	22.0%
	Yes (no health)	17.6%	18.9%	13.2%	25.0%	14.9%	7.4%	20.9%	13.6%	7.7%	26.8%
	Yes (in support text)	58.1%	59.5%	64.5%	57.5%	59.6%	59.3%	46.5%	40.9%	76.9%	51.2%
	No	0.9%	0.0%	1.3%	0.0%	0.0%	3.7%	2.3%	0.0%	0.0%	0.0%
10b) Flood risk management	Yes (health in policy)	2.9%	0.0%	5.3%	2.5%	2.1%	3.7%	2.3%	0.0%	7.7%	2.4%
	Yes (no health)	85.8%	97.3%	78.9%	80.0%	95.7%	81.5%	83.7%	90.9%	76.9%	78.0%
	Yes (in support text)	6.6%	2.7%	6.6%	10.0%	2.1%	7.4%	4.7%	4.5%	15.4%	12.2%
	No	4.6%	0.0%	9.2%	7.5%	0.0%	7.4%	9.3%	0.0%	0.0%	7.3%
10c) Urban heat island and overheating	Yes (health in policy)	2.3%	0.0%	5.4%	0.0%	4.3%	0.0%	2.3%	0.0%	0.0%	2.4%
	Yes (no health)	35.3%	64.9%	36.8%	35.0%	31.9%	29.6%	32.6%	27.3%	23.1%	22.0%
	Yes (in support text)	12.4%	18.9%	15.8%	12.5%	8.5%	14.8%	4.7%	9.1%	15.4%	7.3%
	No	50.0%	16.2%	42.1%	52.5%	55.3%	55.6%	60.5%	63.6%	61.5%	68.3%
10d) Air and noise pollution	Yes (health in policy)	41.6%	32.4%	39.2%	57.5%	44.7%	48.1%	37.2%	54.5%	23.1%	29.3%
	Yes (no health)	26.3%	24.3%	20.3%	17.5%	25.5%	33.3%	30.2%	22.7%	15.4%	43.9%
	Yes (in support text)	26.2%	40.5%	32.4%	15.0%	27.7%	18.5%	14.0%	22.7%	61.5%	22.0%
	No	5.8%	2.7%	8.1%	10.0%	2.1%	0.0%	18.6%	0.0%	0.0%	4.9%
11a) Hot food takeaways	Yes (health	14.2%	27.0%	2.6%	2.5%	8.5%	18.5%	14.0%	9.1%	46.2%	36.6%
	in policy)										
	Yes (no health)	24.0%	21.6%	25.0%	22.5%	36.2%	25.9%	27.9%	9.1%	23.1%	12.2%
	Yes (in support text)	15.9%	37.8%	2.6%	7.5%	6.4%	18.5%	14.0%	36.4%	23.1%	24.4%
	No	46.0%	13.5%	69.7%	67.5%	48.9%	37.0%	48.8%	45.5%	7.7%	29.3%
11b) Food growing	Yes (health in policy)	17.6%	5.4%	11.8%	7.5%	19.1%	33.3%	27.9%	22.7%	23.1%	19.5%
	Yes (no health)	18.8%	29.7%	17.1%	35.0%	12.8%	11.1%	11.6%	13.6%	7.7%	17.1%
	Yes (in support text)	27.2%	51.4%	22.4%	20.0%	14.9%	37.0%	25.6%	27.3%	30.8%	31.7%
	No	36.4%	13.5%	48.7%	37.5%	53.2%	18.5%	34.9%	36.4%	38.5%	31.7%
12. Social and community infrastructure	Yes	95.7%	97.3%	94.7%	97.5%	95.7%	96.3%	93.0%	100.0%	84.6%	95.1%
	No	4.3%	0.0%	5.3%	2.5%	4.3%	3.7%	7.0%	0.0%	15.4%	4.9%
13. Healthy planning frameworks	Yes (in policy)	15.0%	43.2%	3.9%	12.5%	14.9%	11.1%	20.9%	9.1%	15.4%	14.6%
	Yes (in support text)	14.2%	5.4%	13.2%	7.5%	17.0%	7.4%	30.2%	18.2%	23.1%	9.8%
	Yes (no health)	49.1%	37.8%	50.0%	65.0%	44.7%	55.6%	30.2%	72.7%	30.8%	48.8%
	No	21.7%	13.5%	32.9%	15.0%	23.4%	25.9%	18.6%	0.0%	30.8%	26.8%
14. Require health impact assessment	Yes (triggers)	28.9%	43.2%	22.4%	25.0%	36.2%	25.9%	32.6%	13.6%	30.8%	24.4%
	Yes (no triggers)	4.3%	2.7%	1.3%	2.5%	4.3%	3.7%	2.3%	13.6%	0.0%	12.2%
	Yes (in support text)	4.9%	8.1%	0.0%	10.0%	6.4%	0.0%	4.7%	0.0%	23.1%	2.4%
	No	61.8%	45.9%	76.3%	62.5%	53.2%	70.4%	60.5%	72.7%	46.2%	61.0%
S. Supplementary: gender	Yes	10.4%	27.0%	5.3%	7.5%	12.8%	7.4%	2.3%	4.5%	7.7%	17.1%
	No	89.6%	73.0%	94.7%	92.5%	87.2%	92.6%	97.7%	95.5%	92.3%	82.9%
S. Supplementary: mental wellbeing	Yes	59.2%	75.7%	48.7%	50.0%	61.7%	59.3%	55.8%	59.1%	84.6%	58.5%
	No	40.8%	24.3%	51.3%	50.0%	38.3%	40.7%	44.2%	36.4%	15.4%	41.5%
S. Supplementary: crime prevention	Yes	90.5%	97.3%	81.6%	87.5%	95.7%	96.3%	83.7%	95.5%	92.3%	92.7%
	No	9.5%	2.7%	18.4%	12.5%	4.3%	3.7%	16.3%	4.5%	7.7%	7.3%
S. Supplementary: suicide prevention	Yes	1.7%	13.5%	0.0%	2.5%	0.0%	0.0%	0.0%	0.0%	0.0%	0.0%
	No	98.3%	86.5%	100.0%	97.5%	100.0%	100.0%	100.0%	100.0%	100.0%	100.0%

**Table 3:** Comparison of Public Health and Planning Systems Alignment Results.

Review parameter	Response	England (from Chang 2019)	England (from this census)	Change
Total local plans reviewed		322	346	
		*Percentage of local plans reviewed*		
Link to Joint Strategic Needs Assessment (JSNA)	Yes	27%	36.4%	↑
	No	73%	63.6%	↓
Link to Joint Health and Wellbeing Strategy (JHWS)	Yes	23%	29.2%	↑
	No	77%	70.8%	↓
